# Community intervention for cardiovascular disease risk factors in Kalutara, Sri Lanka

**DOI:** 10.1186/s12872-020-01427-y

**Published:** 2020-04-28

**Authors:** L. Gamlath, S. Nandasena, P. de Silva, S. Morrell, C. Linhart, S. Lin, A. Sharpe, S. Nathan, R. Taylor

**Affiliations:** 1 Ministry of Health, Colombo, Sri Lanka; formerly Director, National Institute of Health Sciences, Kalutara, Sri Lanka; 2 Office of the Regional Director of Health Services, Kalutara, Sri Lanka; 3 Ministry of Health, Colombo, Sri Lanka; 4 School of Public Health and Community Medicine, University of NSW, Sydney, Australia

**Keywords:** Cardiovascular disease, Type 2 diabetes, Risk factors, Health promotion, Sri Lanka

## Abstract

**Background:**

The effectiveness of a 2015–17 community intervention to reduce cardiovascular disease (CVD) and type 2 diabetes (T2DM) risk factors is assessed in a Sri Lanka adult population, using a before-and-after study design.

**Methods:**

Four contiguous Public Health Midwife (PHM) areas in Kalutara district (Western Province) were exposed to a Sri Lankan designed community health promotion initiatives (without screening) to lower CVD and T2DM risk factors. Pre- and post-intervention surveys (2014, *n*=1,019; 2017, *n*=908) were of 25–64 year males (M) and females (F) from dissimilar randomly selected clusters (villages or settlements) from PHMs, with probability of selection proportional to population size, followed by household sampling, then individual selection to yield equal-probability samples. Differences in resting blood pressure (BP), fasting plasma glucose (FPG), total cholesterol, body mass index and tobacco smoking, adjusting for cluster sampling, age and socio-economic differences, were examined.

**Results:**

Hypertension prevalence declined from 25% to 16% (F) (*p*<.0001), and 21% to 17% (M). Both mean systolic and diastolic BP declined. T2DM declined from 18% to 13% (F), and 18% to 15% (M), as did mean fasting plasma glucose. Elevated total cholesterol declined from 21% to 15% in women (*p*=0.003) and mean cholesterol declined. Frequency distributions, medians and means of these continuous CVD risk factors shifted to lower levels, and were mostly statistically significant (*p*< 0.05).

**Conclusions:**

Community health promotion can lower key CVD and T2DM risk factors. Lowering tobacco consumption in males and obesity remain challenges in Sri Lanka.

## Background

During the past century Sri Lanka has experienced declines in rates of infectious disease mortality in women, leading to reduction in total female mortality and continued improvement in female life expectancy. By contrast, the absolute decline in infectious disease mortality in men has been accompanied by increased cardiovascular disease (CVD) mortality, leading to a prolonged plateau in male life expectancy [1]. The increasing burden of non-communicable diseases (NCD) in low- and to middle-income countries (LMIC) in the 21st Century, such as Sri Lanka, constitutes an aspect of the epidemiological transition experienced by developed countries half a century previously.

According to the World Health Organization (WHO), such shifts in disease profile require corresponding changes in health care systems, from a focus on infectious diseases to a more comprehensive model that includes NCD prevention and care [2]. Moreover, WHO has advocated a shift from expensive, treatment-focused models toward more affordable preventive and health promotion measures, with less emphasis on the high-risk approach and more stress on a population approach [3, 4].

Three main risk factors for elevated incidence and mortality of atherosclerotic CVD – hypertension, and elevated blood cholesterol and tobacco smoking – were established by cohort studies, particularly the Framingham study, [5] the Seven Countries study, [6] and the British Doctors Study, [7, 8] amongst others. From the 1970s, mortality rates from atherosclerotic CVD in many affected countries declined following elucidation of these risk factors, along with diabetes and elevated plasma glucose, and their causes in diet (excess saturated fat, salt and energy intake), tobacco smoking, and inadequate physical activity with obesity as an important intermediary. This has been further supported by studies of the effects of risk factor reduction in individuals and groups, [9–11] and in whole populations, [12, 13] leading to declines in CVD mortality, with the Finnish North Karelia Project a prominent and successful example [14].

Control of NCDs involves both the population approach involving prevention through decreasing risk factors by health promotion and structural changes directed at the entire population, [4, 15, 16] and the individual high-risk approach involving detection and management of cases at high risk. The WHO update on primary health care (PHC) in 2008 emphasised *inter alia* promotion of healthier lifestyles, [17] exemplified by the “Best Buys” approach. This derives ultimately from the first WHO report on community control of cardiovascular disease published in 1984, [18] and on other work by Geoffrey Rose, [19] aimed at those at moderate and high risk of CVD. In addition, the WHO developed the Package of Essential Non-communicable disease interventions (PEN) approach for developing countries, using high risk CVD detection from testing, and comparison with (hypothetical) regional multi-risk charts, and treatment through primary health care medical facilities.

The PHC setting in LMICs plays an important role in NCD prevention and control [20–22] because of the proximity of PHC and community health care workers to local communities, and their distribution in both rural and urban areas [23, 24]. With the decline in infectious disease and under-nutrition, and the shift of much of premature mortality from < 5 years to adult ages, there is a need to re-orient PHC prevention and control to include the major causes of morbidity and premature mortality. This has changed in many populations to cardiovascular disease, diabetes and other chronic conditions in adults, while preserving important maternal and child health initiatives, and nutrition and environmental health priorities.

In Sri Lanka, several risk factor prevalence sample surveys for NCD have been undertaken, [25, 26] although comparisons are difficult because of variations in age groups and rural/urban compositions of those surveyed. Tobacco smoking has changed little (around 30% in men, <1% in women), mean BMI appears to be increasing in both sexes, and blood pressure (BP) and hypertension (HT) also appear to be increasing, with HT prevalence recently estimated at 25% [27]. Measures of fasting blood glucose (FBG) and prevalence of T2DM (8.5–24%) have been highly variable, [28] and are particularly sensitive to the rural/urban composition of the survey samples.

Current programs to address CVD risk factors in Sri Lanka are influenced by the development of Healthy Lifestyle Centres (HLC), initiated by the Ministry of Health (MoH) and the Japan International Cooperation Agency (JICA) from 2008–2013. HLCs numbered 826 by 2016, [29] and (anecdotally) 900 by 2018 [30]. Men and women aged 35–65 years are encouraged to attend, and following self-referral are screened for BP, FBG and body mass index (BMI), with lifestyle assessment and counselling provided on physical exercise, diet, tobacco use and alcohol consumption. No baseline studies were performed in areas prior to the HLC initiative, and current monitoring is limited to self-presenters. Comparisons of HLC attendees with the broader population are inconclusive, [29] and the effectiveness of the HLC approach based on voluntary counselling and testing remains unevaluated. The HLCs have considerably lower male than female attendance rates [31].

A large proportion of resources for controlling NCD in Sri Lanka, under the 2010 NCD strategic framework, has been expended on secondary and tertiary care, [31] including: increased NCD medication supplies in primary care facilities; and increased specialised facilities and numbers of doctors and specialists with training in NCD diagnosis and treatment. In contrast, health resources committed to NCD prevention have not been as fully implemented, including: promotion of population-wide healthy lifestyle programmes; media-advocacy campaigns to reduce intake of salt, sugar, fat; and implementation of the healthy school canteen policy. Physician informants to the report [31] also noted that “most of the NCD money was not directed to evidence-based ‘best buys’ for reducing and managing NCDs”.

To examine if a population-based health promotion approach is feasible and could be successful in decreasing NCD risk factors, a population intervention in the Western Province of Sri Lanka was conducted from 2014 to 2017 to reduce risk factors for atherosclerotic CVD in areas without HLCs, using a Sri Lankan designed community health promotion approach through primary health care and CHWs. The objective of the intervention was to shift population frequency distributions of CVD risk factors to lower levels, and to reduce means and prevalences of these CVD risk factors.

## Methods

### Health promotion intervention

The campaign of community health promotion used established networks of primary health care workers (PHCW), and was initiated in 2015 after the first Kalutara risk factor survey (2014). The community interventions were designed by senior local investigators (LG, SN1 and PdeS), based on an extensive review of pilot CVD prevention programs in Sri Lanka, and on their detailed knowledge of previous successful community engagement in healthcare in the country. No HLCs were located within the study area during the study period. Public health midwives (PHM) were enlisted to communicate with women concerning the need to reduce CVD, T2DM, and risk factors. PHMs organised meetings in venues commonly used for public gatherings. Suitable women were invited to a community clinic centre and involved in an awareness program to reduce dietary salt, sugar and animal fat, and promote physical activity, run by local medical officers, nurses and public health inspectors.

The participating women acted as change agents to influence others in the community, with whom they could easily communicate, including other opinion leaders [32]. Each of the four PHM areas was instructed to recruit at least 20 change agents. As the PHM areas varied in size, between 80 and 100 change agents in total were recruited. Women were consciously selected for recruiting women as change agents since in Sri Lanka they are more involved than men in shopping, cooking, child and extended family care, household planning, and similar activities. Education sessions (> 10) led by these women were then held, with 20–40 participants in each session.

NCD prevention messages were integrated into PHM home visit services and into fortnightly PHM outreach community clinics routinely conducted for immunisation, antenatal care, children’s growth monitoring, and similar activities. PHMs spend at least 15 days per month on home visits and 3–4 days in the clinics in their area. Regular monthly in-service training programs for PHMs now include sessions pertaining to NCD.

As part of the intervention, a health and lifestyle carnival was held in Kalutara on 4 July 2015 and attended by approximately 2,500 people of all ages. The carnival showcased various community activities to promote healthy lifestyles. Stalls demonstrated cooking of different traditional foods, and recipes were given to attendees. Street theatre and exercise demonstrations were featured, and plays performed by pre-schoolchildren promoting healthy lifestyles. BP and anthropometric measurements were available on request.

The intervention was implemented continuously over 2015–17. Throughout the intervention health messages were sent frequently to the entire community via different media, primarily print and face-to-face. Particular focus was placed on women to take up local sports (e.g., *elle*, a Sri Lankan game similar to baseball). Health promotion messages were kept simple and directed specifically at lowering proximate NCD risk factors such as reduction of added salt in cooking, lessening dietary intake of animal fat, quitting tobacco smoking, etc. Much of the preventive interventions were implemented through and by women who are particularly concerned with diet and food preparation.

The health promotion intervention involved trained community health workers, utilising the existing primary healthcare network, along with influential members of the local community, who provided face-to-face verbal dissemination of preventive information both to individuals (e.g. in clinical settings, opportunistically or purposively) and to groups (e.g. in purposive public gatherings); provision of printed material on healthy eating (especially reduction of salt and saturated fat intake), and on regular physical activity; and inserting prevention messages into entertainment settings (e.g. the carnival). This approach aimed to maximise the reach and appeal of the health promotion interventions across different population segments.

### Monitoring of risk factors

#### Sampling

For the community health promotion interventions, four contiguous PHM areas (estimated 25–64 year population at the 2012 census of 8,100) were selected as the study site from within the Kalutara Health area. Identical random sampling methods were employed for both surveys to produce dissimilar clusters and individuals, but to ensure representative and comparable samples. To monitor the consequences of the intervention, village or settlement clusters were randomly selected from each PHM area, with a probability of selection of each proportional to population size, followed by household sampling, then individual selection (Kish grid method), [33] to yield equal-probability samples of approximately 1,000 adults for the 2014 and 2017 surveys. For each survey, 8 clusters (of 24 available) were randomly selected from PHM area Bombuwala I (2012 Census adults 25-64 years =2,021, sample =250); 8 (of 19) from Bombuwala II (2012 Census adults=2,106, sample =250); 6 (of 21) from Gamgoda (2012 Census adults =1,307, sample =200); and 10 (of 10) from Nagoda (2012 Census adults =2,577, sample =300). Actual samples were: 2014 survey, 1,019 (sampling fraction 12.7%), and 2017 survey, 908 (sampling fraction 11.3%), based on 2012 census estimates for adults 25-64 years resident in the 4 PHM areas. Subjects examined in both surveys were excluded from analyses (*n*=23). Samples were randomly selected to be representative of the study population for the before-and-after comparison.

#### Data Collection

Data were collected as in the first survey, where further details can be found [26]. On the day preceding the survey, participants were recruited and advised to fast overnight. Questionnaires using a modified WHO STEPS survey instrument were administered by trained staff (Step 1), with physical (Step 2), and biochemical measurements (Step 3) taken. The WHO questionnaire was administered in Sinhalese (its translation from English was verified by back-translation by independent translators). Height, weight, and waist circumference were measured once. Obesity was defined as body mass index (BMI) $\geqslant $27.5 kg/m^2^ (Asian) (WHO standard) [34].

Respondents were seated for 10 minutes before three blood pressure readings were taken, with 3–5 minute intervals between measurements. Mean systolic blood pressure *SBP* and diastolic blood pressure *DBP* were calculated from the first two BP readings if the difference between them was < 10 mm Hg, or from the second and third readings if the difference was greater. Hypertension was signified by SBP $\geqslant $140 mm Hg or DBP $\geqslant $90 mm Hg or if the participant had been taking anti-hypertensive medication in the previous two weeks [35].

In the 2014 baseline survey, fasting plasma glucose *FPG* was measured using a Roche ACCU-CHEK glucose meter with supplied test strips, calibrated to provide plasma glucose equivalent measurements from capillary whole blood samples [26]. In the follow-up survey (2017) glucose was measured in whole capillary blood samples by a Major Bio II glucose meter with supplied test strips, uncalibrated to plasma levels, so the readings were multiplied by 1.11 to correspond to a plasma concentration reading [36]. To allow for systematic differences found between glucose meters, FPG readings of the second 2017 survey were inflated by a further factor of 1.07. T2DM was defined as FPG $\geqslant $7.0 mmol/L or taking medication for T2DM in the previous 2 weeks [37].

Total plasma cholesterol was measured on capillary whole blood by supplied test strips using a Roche Accutrend Plus cholesterol meter in the 2014 survey, and a Mission Ultra cholesterol meter (Acon Labs), in 2017. Elevated (‘high’) total plasma cholesterol was defined as > 6.2 mmol/L (based on current clinical practice [38]), consistent with Rose [1992], [39] or taking medication for high cholesterol in the previous 2 weeks.

Participants with elevated absolute CVD risk factor results ($\geqslant $30% probability of a CVD event over 10 years) according to WHO multi-risk charts applicable to Sri Lanka [40] were advised according to Sri Lanka MoH guidelines to attend the local health facility for follow-up. All data were recorded using a standardized survey form, coded and entered into EpiData software [41]. Data were validated by re-checking with the original survey forms. Ethics approval was obtained from the National Institute of Health Sciences Ethics Review Committee prior to commencement of the study. All participants provided informed verbal consent to participate in the survey before any measurements were obtained.

#### Analysis

Inter-survey differences in mean in systolic and diastolic blood pressures, fasting plasma glucose, total cholesterol and body mass index are compared using t-tests. Inter-survey differences in prevalences of HT, T2DM/high FPG, high total cholesterol and obesity are compared using chi-squared statistics, and odds ratios estimated from logistic regression. Analyses are adjusted for cluster sampling, and age, using the combined survey age strata as the weighting standard, or from regression based on individual data. Age differences between surveys are minimal (Table 1), but age is included as a universal confounder. Means and prevalence ratios are additionally adjusted for income and education (using linear and logistic regression) since the baseline study showed some socio-economic associations with CVD risk factors [26]. Differences in median values were tested by the two-sample median test, and differences in frequency distributions by the Kolmogorov-Smirnov test, using SAS (version 9.4).

**Table 1:** Mean age, systolic and diastolic blood pressures, fasting plasma glucose, total cholesterol and body mass index in adults aged 25–64 years, by sex and survey, Kalutara, Sri Lanka

**Risk Factor**	**Sex**	**First Survey (2014)**	**Second Survey (2017)**	**Decrease**	***P****-value*
Age (yr)	F	43.0 (41.9 – 44.2)	41.7 (40.5 – 43.0)	1.31 (2.70 – +0.08)	0.0641
	M	41.6 (40.4 – 42.7)	41.5 (39.9 – 43.1)	0.08 (1.78 – +1.62)	0.9266
Systolic blood	F	123.9 (121.9 – 125.8)	119.8 (118.3 – 121.2)	**4.14 (6.31 – 1.96)**	**0.0003**
pressure (mmHg)			Adjusted:^a^	**3.19 (5.04 – 1.34)**	**0.0010**
			Adjusted:^b^	**2.83 (4.54 – 1.12)**	**0.0015**
	M	125.2 (123.3 – 127.1)	124.3 (121.7 – 126.9)	0.88 (4.04 – +2.30)	0.5814
			Adjusted:^a^	0.84 (3.98 – +2.29)	0.5918
			Adjusted:^b^	2.08 (4.90 – +0.74)	0.1447
Diastolic blood	F	73.5 (72.6 – 74.4)	71.2 (70.4 – 71.9)	**2.32 (3.36 – 1.28)**	**<.0001**
pressure (mmHg)			Adjusted:^a^	**2.03 (2.93 – 1.13)**	**<.0001**
			Adjusted:^b^	**2.01 (2.94 – 1.08)**	**0.0001**
	M	72.4 (71.2 – 73.6)	71.2 (69.5 – 72.8)	1.24 (3.16 – +0.68)	0.2019
			Adjusted:^a^	1.22 (3.20 – +0.76)	0.2226
			Adjusted:^b^	**2.04 (4.00 – 0.07)**	**0.0431**
Fasting plasma	F	6.39 (6.21 – 6.57)	5.68 (5.51 – 5.85)	**0.71 (0.95 – 0.47)**	**<.0001**
glucose (mmol/L)			Adjusted:^a^	**0.67 (0.91 – 0.43)**	**<.0001**
			Adjusted:^b^	**0.63 (0.88 – 0.38)**	**<.0001**
	M	6.34 (6.12 – 6.57)	5.74 (5.57 – 5.92)	**0.60 (0.88 – 0.32)**	**<.0001**
			Adjusted:^a^	**0.60 (0.88 – 0.31)**	**<.0001**
			Adjusted:^b^	**0.72 (1.06 – 0.39)**	**<.0001**
Total	F	5.56 (5.38 – 5.74)	4.91 (4.82 – 4.99)	**0.66 (0.86 – 0.45)**	**<.0001**
cholesterol (mmol/L)			Adjusted:^a^	**0.63 (0.84 – 0.42)**	**<.0001**
			Adjusted:^b^	**0.60 (0.84 – 0.34)**	**<.0001**
	M	5.50 (5.34 – 5.66)	5.03 (4.91 – 5.15)	**0.47 (0.66 – 0.29)**	**<.0001**
			Adjusted:^a^	**0.47 (0.66 – 0.28)**	**<.0001**
			Adjusted:^b^	**0.50 (0.75 – 0.25)**	**0.0002**
Body mass	F	24.8 (24.5 – 25.1)	25.4 (25.0 – 25.7)	**+0.58 (+0.72 – +1.08)**	**0.0256**
index (kg/m^2^)			Adjusted:^a^	**+0.59 (+0.64 – +1.11)**	**0.0284**
			Adjusted:^b^	+0.40 (0.11 – +0.91)	0.1180
	M	23.1 (22.6 – 23.5)	23.7 (23.0 – 24.3)	+0.57 (0.12 – +1.25)	0.1051
			Adjusted:^a^	+0.56 (0.11 – +1.23)	0.0980
			Adjusted:^b^	+0.06 (0.67 – +0.79)	0.8628

M = males; *F*= females. First survey 2014 (*n*= 412 M, 606 F); Second survey 2017 (*n*= 272 M, 613 F)

Means and 95% CIs adjusted for cluster sampling (by village/settlement)

^a^Mean difference adjusted for age and cluster sampling by linear regression

^b^Mean difference adjusted for age, cluster sampling, education level and income by linear regression

**Bolded results:** significant at p ≤0.05.

Note: analyses exclude subjects who participated in both surveys (*n*=23)

## Results

After excluding those participating in both surveys (*n*=23), there are 1,018 from the first survey (606 women, 412 men), and 885 from the second survey (613 women, 272 men). There are minimal, non-significant differences between mean ages in the two surveys (Table 1). Some differences in the socio-economic status of the samples were evident, where the respondents in the second survey tended to have higher income and education levels (see Appendix, Table 4). Despite these differences, the results for high BP, FPG and total cholesterol were similar across these socioeconomic strata. (Appendix, Table 5). Survey differences in income and education levels, along with age, were controlled for in statistical analyses.

**Table 2:** Prevalences (%), with prevalence and odds ratios of hypertension, type 2 diabetes, high total cholesterol, obesity and smoking status by survey, adults aged 25–64 years, males and females, Kalutara, Sri Lanka

**Risk Factor**	**Sex**	**First**	**Second**	**Second:First survey**	***P****-value*
		**Survey**	**Survey**	**Prevalence/Odds ratio**	
Hypertension	F	24.7	16.1	**0.65 (0.51 – 0.84)**	**0.0002**
			Adjusted OR^a^	**0.53 (0.40 – 0.70)**	**<.0001**
			Adjusted OR^b^	**0.56 (0.42 – 0.75)**	**0.0002**
	M	21.1	16.6	0.79 (0.56 – 1.10)	0.1557
			Adjusted OR^a^	0.75 (0.49 – 1.15)	0.1816
			Adjusted OR^b^	**0.63 (0.40 – 0.98)**	**0.0410**
Diabetes	F	18.2	13.5	0.74 (0.53– 1.04)	0.0859
			Adjusted OR^a^	0.71 (0.48 – 1.03)	0.0721
			Adjusted OR^b^	0.70 (0.48 – 1.04)	0.0754
	M	17.5	15.2	0.87 (0.55 – 1.38)	0.5380
			Adjusted OR^a^	0.84 (0.48 – 1.46)	0.5264
			Adjusted OR^b^	0.70 (0.40 – 1.23)	0.2112
High total cholesterol	F	21.5	15.2	**0.71 (0.58 – 0.87)**	**0.0011**
			Adjusted OR^a^	**0.62 (0.49 – 0.79)**	**0.0002**
			Adjusted OR^b^	**0.65 (0.49 – 0.87)**	**0.0040**
	M	18.9	20.1	1.07 (0.79 – 1.45)	0.6740
			Adjusted OR^a^	1.05 (0.73 – 1.51)	0.7880
			Adjusted OR^b^	0.95 (0.65 – 1.39)	0.7782
Obesity	F	25.6	28.7	1.12 (0.95 – 1.32)	0.1744
			Adjusted OR^a^	1.18 (0.96 – 1.46)	0.1220
			Adjusted OR^b^	1.13 (0.89 – 1.44)	0.3015
	M	11.3	14.4	1.27 (0.95 – 1.71)	0.1129
			Adjusted OR^a^	1.35 (0.96 – 1.90)	0.0874
			Adjusted OR^b^	1.19 (0.82 – 1.72)	0.3656
Current smoker	F	0.29	0.14	0.49 (0.04 – 5.56)	0.5410
			Adjusted OR^a^	0.55 (0.05 – 5.56)	0.6109
			Adjusted OR^b^	-	-
	M	29.1	27.7	0.95 (0.71 – 1.27)	0.7301
			Adjusted OR^a^	0.97 (0.64 – 1.47)	0.8891
			Adjusted OR^b^	1.19 (0.84 – 1.69)	0.3172
Daily smoker	F	0.29	0.14	0.49 (0.04 – 5.56)	0.5410
			Adjusted OR^a^	0.55 (0.05 – 5.56)	0.6109
			Adjusted OR^b^	-	-
	M	20.4	19.9	0.98 (0.65 – 1.47)	0.9146
			Adjusted OR^a^	1.01 (0.60 – 1.70)	0.9725
			Adjusted OR^b^	1.32 (0.79 – 2.21)	0.2865

M = males; *F*= females. First survey 2014 (*n*= 412 M, 606 F); Second survey 2017 (*n*= 272 M, 613 F)

Prevalence ratio adjusted for cluster sampling (by village/settlement)

^a^Odds ratios (ORs) adjusted for age and cluster sampling

^b^Odds ratios (ORs) adjusted for age, cluster sampling, income and education levels

*Hypertension*: SBP$\geqslant $140 mmHg or DBP$\geqslant $90 mmHg or taking medication for high BP

*Diabetes/High FPG*: Fasting plasma glucose$\geqslant $7 mmol/l or taking medication for T2DM

*High cholesterol*: total cholesterol > 6.2 mmol/l or taking medication for high cholesterol

*Obesity*: body mass index$\geqslant $27.5 kg/m ^2^

*Smoking*: smoking tobacco – manufactured or hand-rolled cigarettes, cigars or pipe

**Bolded results:** significant at p ≤0.05.

Note: analyses exclude subjects who participated in both surveys (*n*=23)

**Table 3:** Medians of systolic and diastolic blood pressures, fasting plasma glucose, total cholesterol and body mass index in adults aged 25–64 years, by sex and survey, Kalutara, Sri Lanka

**Risk Factor**	**Sex**	**First**	**Second**	**Decrease**	***P****-value of*	***P****-value of*
		**Survey**	**Survey**		**median**	**distribution**
		**(2014)**	**(2017)**		**difference**^a^	**difference**^b^
Systolic blood	F	119.5	118.0	**1.50**	**0.0482**	**0.0068**
pressure (mmHg)	M	124.0	122.5	1.50	0.1415	0.2249
Diastolic blood	F	73.0	70.0	**3.00**	**0.0004**	**0.0023**
pressure (mmHg)	M	72.0	70.0	**2.00**	**0.0314**	0.1590
Fasting plasma	F	5.89	5.23	**0.29**	**<.0001**	**<.0001**
glucose (mmol/L)	M	5.89	5.30	**0.30**	**<.0001**	**<.0001**
Total	F	5.35	4.76	**0.59**	**<.0001**	**<.0001**
cholesterol (mmol/L)	M	5.33	4.84	**0.49**	**<.0001**	**<.0001**
Body mass	F	24.6	25.5	**+0.98**	**0.0099**	**0.0160**
index (kg/m^2^)	M	23.1	23.2	+0.13	0.8514	0.9387

M = males; *F*= females

First survey 2014 (*n*= 412 M, 606 F); Second survey 2017 (*n*= 272 M, 613 F)

^a^Median differences are unadjusted and tested by two-sample median test

^b^Distribution differences (unadjusted) tested by Kolmogorov-Smirnov test

**Bolded results:** significant at p ≤0.05

Note: analyses exclude subjects who participated in both surveys (*n*=23)

**Table 4:** Proportions (%) of respondents by sex, age group, income and education level by survey, adults aged 25–64 years, males and females, Kalutara, Sri Lanka

**Socio-demographic**	**Females**		**Males**	
**Factor**	**First Survey**	**Second Survey**	**First Survey**	**Second Survey**
	**(n=606)**	**(n=412)**	**(n=613)**	**(n=272)**
Age group (yr)				
25–29	8.2	9.0	8.7	8.8
30–34	16.3	16.3	18.7	14.3
35–39	15.8	22.2	21.4	22.1
40–44	17.0	17.0	17.0	22.4
45–49	15.0	13.2	12.1	11.4
50–54	10.7	9.5	8.0	8.8
55–59	9.6	8.0	8.7	8.5
60–64	7.4	4.9	5.3	3.7
Education Level (yrs)				
< 6	13.2	6.0	11.7	4.4
6–10	37.9	36.4	39.8	31.6
> 10	48.9	57.6	48.5	64.0
Income level (LKR)				
< 20,000	54.0	27.2	50.3	18.8
20–30,000	27.8	32.2	32.8	41.9
> 30,000	18.2	40.6	16.9	39.3

Note: analyses exclude subjects who participated in both surveys (*n*=23)

**Table 5:** Odds ratios (age and cluster adjusted, second/first survey), of hypertension, type 2 diabetes, high total cholesterol, by income and education levels, adults aged 25–64 years, males and females, Kalutara, Sri Lanka

**Risk Factor**	**Sex**	**Education (yr)/**	**Odds ratio**^a^	***P****-value*
		**Income (LKR)**	**second/first**	
		**level**	**survey**	
Hypertension^b^	F	≤5 yr	0.49	0.1778
		6–10	0.47	0.0029
		> 10	0.63	0.0012
	M	≤5	0.54	0.5925
		6–10	0.55	0.0920
		> 10	0.79	0.3393
	F	≤20,000 LKR	0.44	<.0001
		20–30,000	0.59	0.0368
		> 30,000	0.77	0.2623
	M	≤20,000	0.35	0.0350
		20–30,000	0.75	0.3525
		> 30,000	0.79	0.5791
High Fasting	F	≤5	0.69	0.4278
Plasma Glucose^c^		6–10	0.66	0.0732
		> 10	0.70	0.3130
	M	≤5	-	-
		6–10	0.83	0.6242
		> 10	0.82	0.5595
	F	≤20,000	1.05	0.7720
		20–30,000	0.61	0.1987
		> 30,000	0.48	0.0376
	M	≤20,000	0.97	0.9535
		20–30,000	0.65	0.2216
		> 30,000	0.69	0.3065
High Fasting	F	≤5	0.72	0.2973
Cholesterol^d^		6–10	0.50	0.0039
		> 10	0.69	0.0816
	M	≤5	0.68	0.6042
		6–10	0.89	0.7118
		> 10	1.17	0.5358
	F	≤20,000	0.78	0.1723
		20–30,000	0.46	0.0097
		> 30,000	0.75	0.3796
	M	≤20,000	0.37	0.1895
		20–30,000	0.77	0.3794
		> 30,000	0.91	0.7752

M = males; *F*= females. First survey 2014 (*n*= 412 M, 606 F); Second survey 2017 (*n*= 272 M, 613 F)

^a^Odds ratios (ORs) adjusted for age and cluster (village) sampling

^b^*Hypertension*: SBP$\geqslant $140 mmHg or DBP$\geqslant $90 mmHg or taking medication for high BP

^c^*Diabetes/High FPG*: Fasting plasma glucose$\geqslant $7 mmol/l or taking medication for T2DM

^d^*High cholesterol*: total cholesterol > 6.2 mmol/l or taking medication for high cholesterol

Note: analyses exclude subjects who participated in both surveys (*n*=23)

### Blood pressure and hypertension

Among women in the second survey, mean SBP was 120 compared to 124 mmHg in the first survey. After adjusting for sampling design effects, this difference was − 4.14 mmHg; after additional adjustment for age, the difference was − 3.2 mmHg (*p*=0.0010), and − 2.8 mmHg after further adjustment for income and education levels (*p*=0.0015) (Table 1). Mean female DBP in the second survey was 71 compared to 74 mmHg in the first survey. The cluster- and age-adjusted difference of − 2.0 mmHg was statistically significant (*p*<.0001), with a similar result after additional adjustment for income and education level. Men showed less decline in mean SBP (0.9 mmHg), but after adjustment for age, education and income the decline was 2.1 mmHg (*p*> 0.05); similarly for DBP, the unadjusted decline of 1.2 mmHg in men and was 2.0 mmHg after adjustment for income and education levels, but was statistically significant (*p*=0.0431).

Between the two surveys, HT prevalence (SBP$\geqslant $140 mm Hg or DBP$\geqslant $90 mm Hg or taking anti-hypertensive medication [35]) declined from 25% to 16% in women, with a prevalence ratio of 0.65 adjusted for age and cluster sampling (*p*<.0001); and from 21% to 17% in men with a prevalence ratio of 0.79 (*p*> 0.05) (Table 2). Further adjustment for income and education using logistic regression gave odds ratios for HT in women of 0.56 (*p*=0.0002) and 0.63 (*p*=0.0410) for men, both statistically significant.

**Fig. 1:**
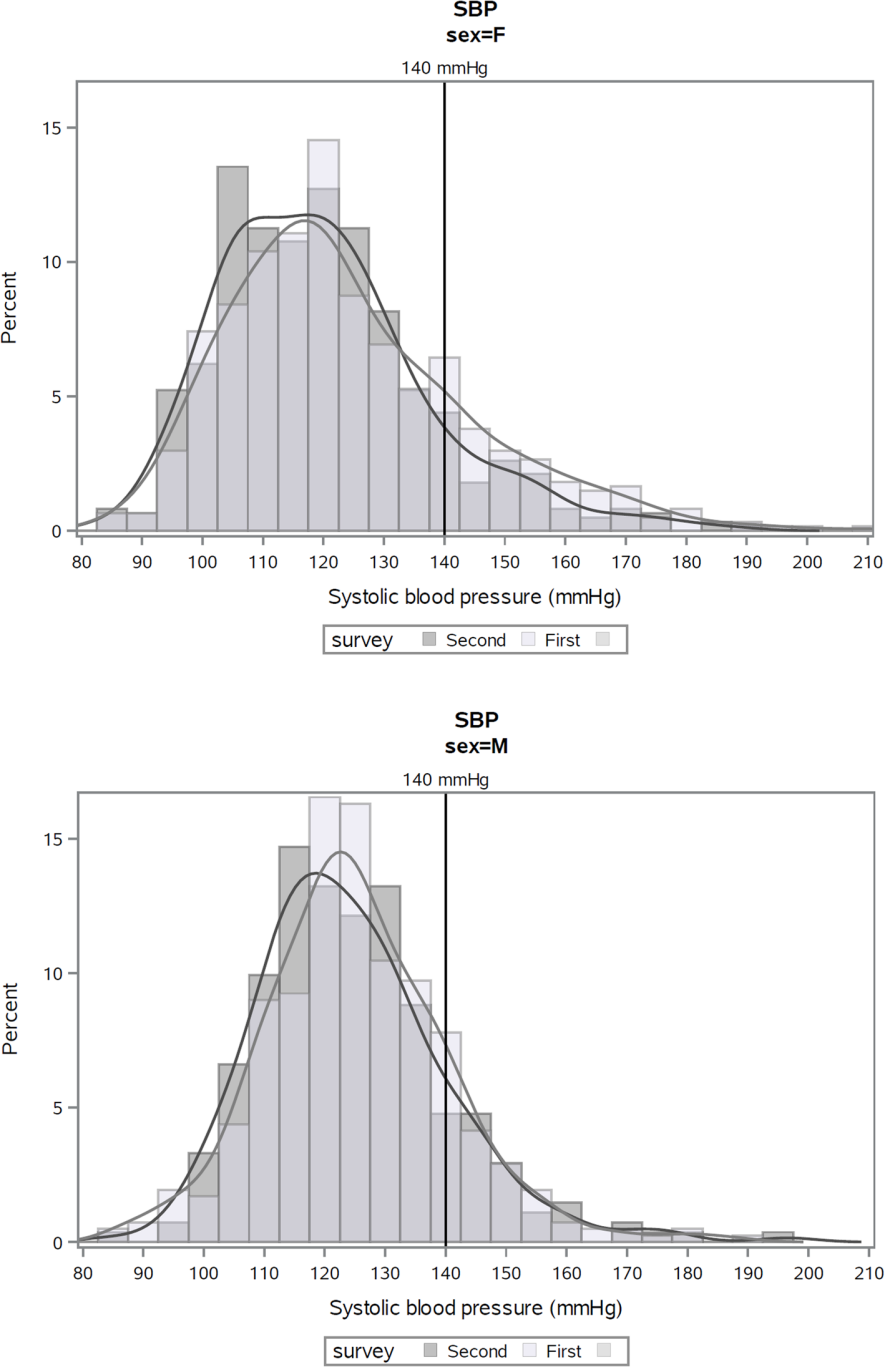
Distribution of systolic blood pressure (SBP) at first and second risk factor surveys, Kalutara adults aged 25–64 years. Note: analyses exclude subjects who participated in both surveys (*n*=23)

**Fig. 2:**
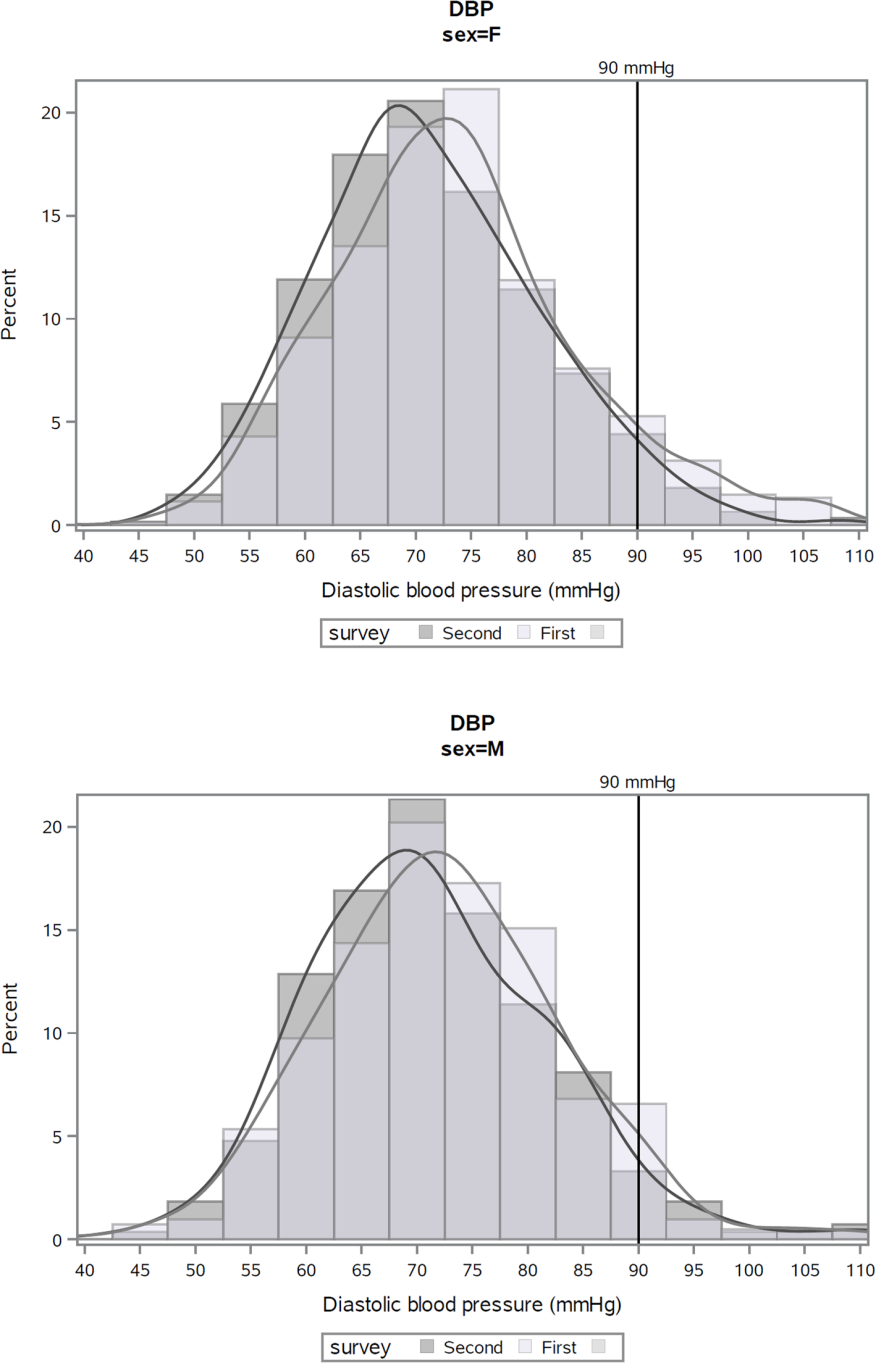
Distribution of diastolic blood pressure (SBP) at first and second risk factor surveys, Kalutara adults aged 25–64 years. Note: analyses exclude subjects who participated in both surveys (*n*=23)

In women, the lower BP frequency distributions in the second survey compared to the first were significant (SBP *p*=0.0068; DBP *p*=0.0023), as well as the lower medians for SBP (*p*=0.0482) and DBP (*p*=0.0004). In men, frequency distributions and medians were lower in the second survey compared to the first, but only the difference in median DBP was statistically significant (*p*=0.0314) (see Table 3 and Figures 1 and 2 in Appendix).

### Fasting plasma glucose and diabetes

Among women in the first survey, mean FPG was 6.39 compared to 5.68 mmol/L in the second survey; in men mean FPG was 6.34 compared to 5.74 mmol/L (Table 1). After adjusting for sampling design effects, age, income and education, the mean differences in FPG levels were lower by − 0.63 mmol/l (*p*<.0001) in women and − 0.72 mmol/l (*p*<.0001) in men.

Prevalence of high FPG/T2DM (T2DM was defined as FPG$\geqslant $7.0 mmol/L or taking medication for T2DM [37]) declined from 18% in the first survey to 14% in the second in women, with a clustering-sampling adjusted prevalence ratio of 0.71 (*p*=0.0011); and from 18% to 15% in men with a prevalence ratio of 0.87 (*p*=0.5380) (Table 2). With additional adjustment for age, income and education level, the OR of high FPG/T2DM was 0.70 in women (*p*=0.0754) and 0.70 in men (*p*=0.2112).

**Fig. 3:**
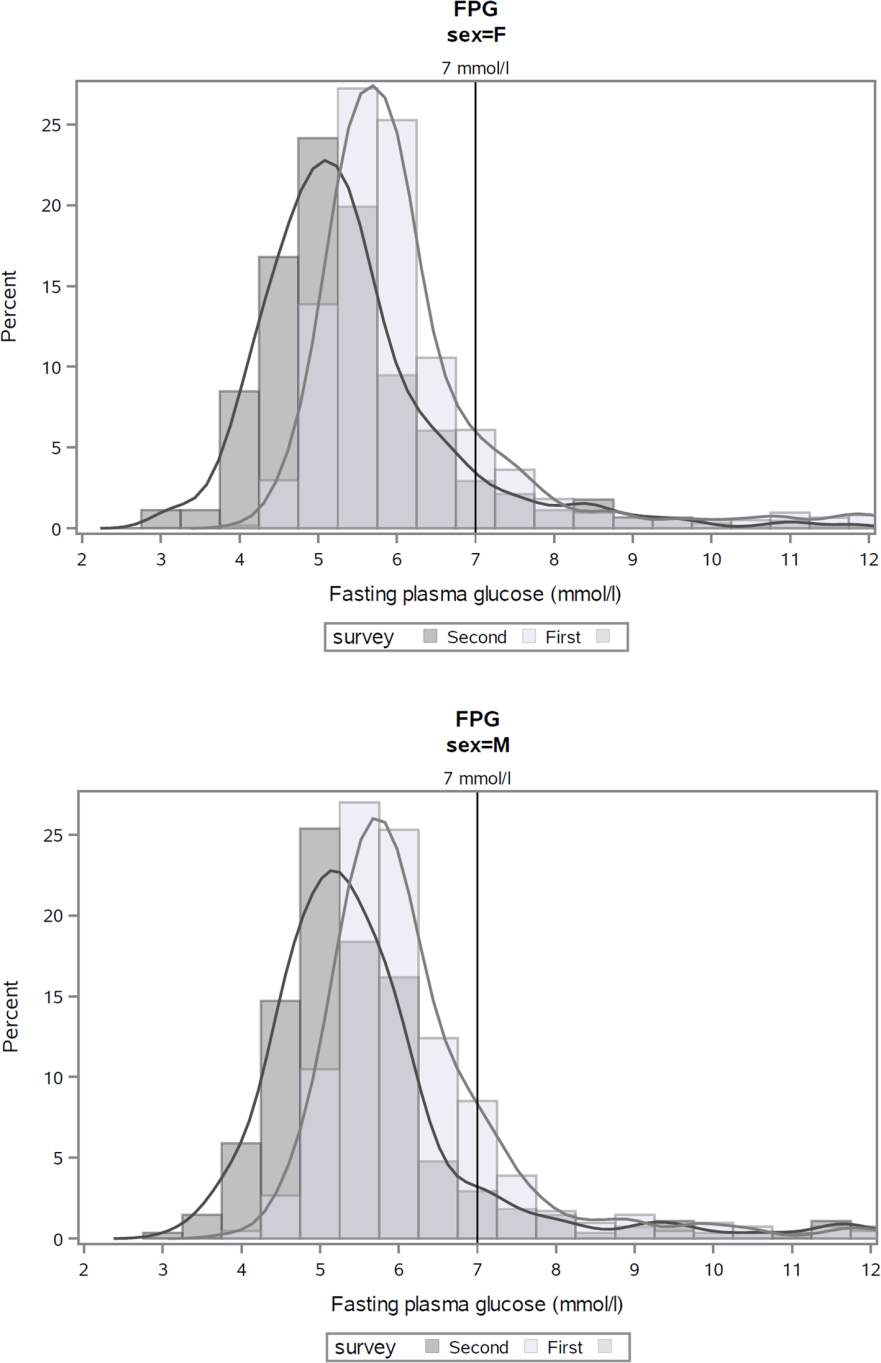
Distribution of fasting plasma glucose levels at first and second risk factor surveys, Kalutara adults aged 25–64 years. Note: analyses exclude subjects who participated in both surveys (*n*=23)

Comparison of the two surveys showed a clear shift in the FPG distributions to lower values in both women and men (Figure 3 in Appendix). The differences in the frequency distributions and medians of FPG in women and men were significant (*p*<.0001) (Table 3).

### Blood cholesterol

Significantly lower mean total cholesterol was found in the second compared to the first survey for both sexes; by − 0.47 mmol/l (men) and − 0.66 mmol/l (women) (both *p*<.0001), adjusted for cluster sampling; and by 0.50 mmol/l and 0.60 mmol/l respectively when further adjusted for income and education level (*p*=0.0002,<.0001 respectively, Table 1).

Prevalence of elevated total blood cholesterol (> 6.2 mmol/L or taking medication for high cholesterol [38, 39]) declined in women from 21% to 15% from the first to the second survey with a cluster-sampling adjusted prevalence ratio of 0.71 (*p*=0.0011), but without significant change in men (19% to 20%, prevalence ratio = 1.07, *p*> 0.05) (Table 2). After also adjusting for age, income and education level, the OR for women was 0.65 (*p*=0.0040), and for men was 0.95 (*p*> 0.05).

**Fig. 4:**
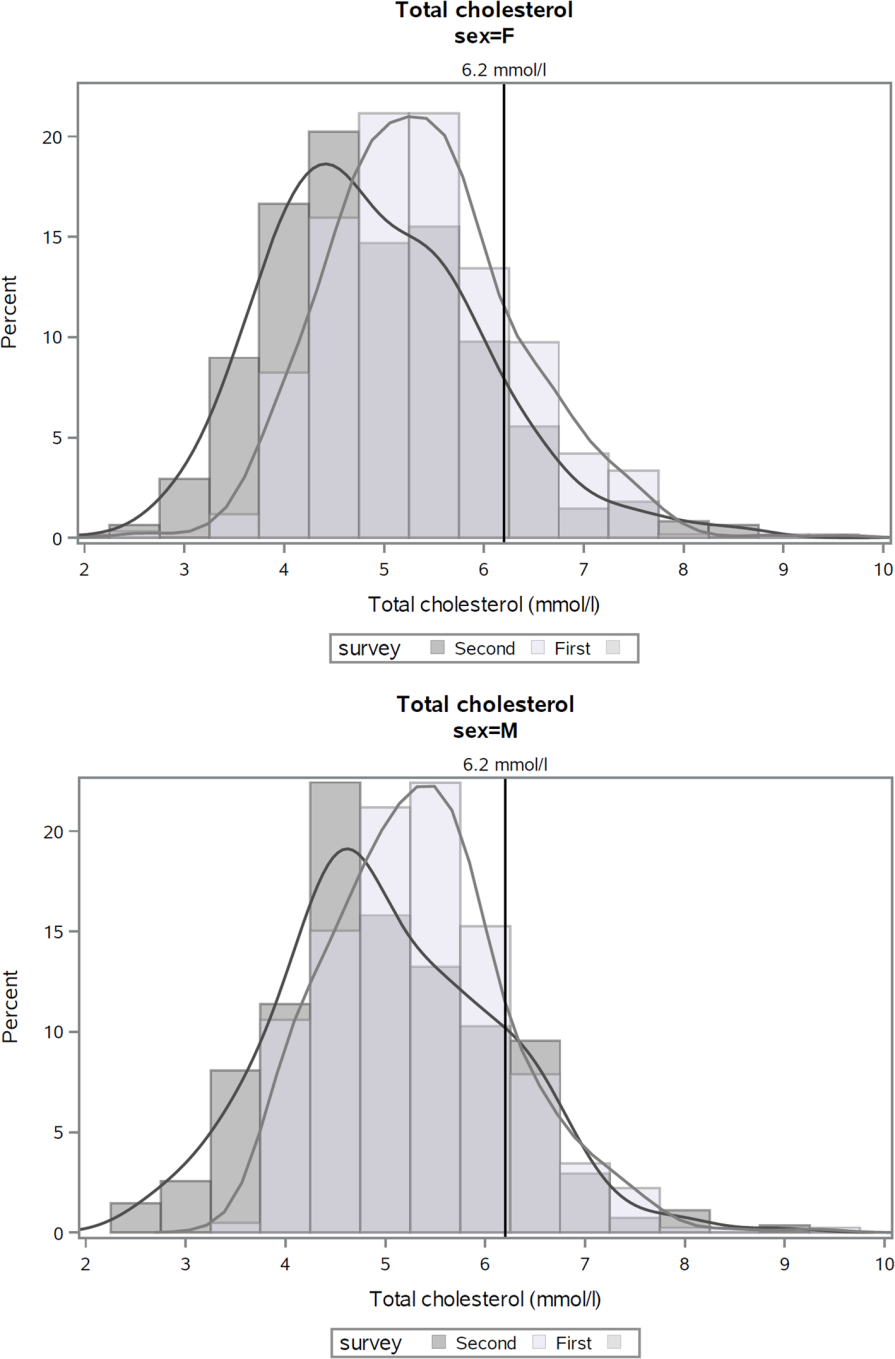
Distribution of total cholesterol at first and second risk factor surveys, Kalutara adults aged 25–64 years. Note: analyses exclude subjects who participated in both surveys (*n*=23)

The frequency distributions of total blood cholesterol in both men and women showed lower values in the second survey compared to the first, and were significantly different (*p*<.0001) for both sexes (Table 3 and Figure 4 in Appendix).

### Body mass index and obesity

The slightly higher (unadjusted) mean and median BMI in both sexes in the second compared to the first survey were no longer statistically significantly different (in women) when adjusted for income and education. For men, slightly higher mean and median BMI at the second survey was not statistically significant, unadjusted or adjusted. The higher obesity odds ratios and prevalences in the second survey were not statistically significant.

### Tobacco smoking

Prevalence of current tobacco smoking of < 1% in women and 28–29% in men were similar in each survey, and not statistically significantly different.

## Discussion

This study assessed the effectiveness of a Sri Lankan designed and implemented health promotion initiative to reduce risk factors for CVD, implemented continuously over 2015–17 targeting an adult population aged 25–64 years in a part of Kalutara District in the Western Province of Sri Lanka. The intervention aimed to alter diet (saturated fat, salt, energy), increase physical activity, and reduce tobacco smoking; and was assessed by measurement of CVD risk factors by mini-STEPS surveys of different subjects in 2014 prior to the intervention, [26] and after three years of implementation, in 2017. The measurable objective of the intervention was to shift frequency distributions of CVD risk factors to reduce numbers in the population at high and moderate risk of CVD (since risk is continuous). Considerable CVD cases and deaths emanate from those at moderate risk (below accepted diagnostic high-risk cut-offs), as well as from those at high risk whose numbers in the population are much fewer. This observation was made by Geoffrey Rose, [19] and incorporated in the first WHO Expert Committee on control of CVD in 1984 [18].

Given its limited resources, Sri Lanka has an enviable history of a strong and effective primary health care network. Through most of last century Sri Lanka was able to significantly reduce infant and child mortality [42] and maternal mortality, [43] using primary care midwifery. Although PHMs still play an important role in antenatal care and pregnancy risk assessment, almost all pregnant women now deliver in hospital. Consequently, PHM roles have been broadened to encompass a life course approach to ongoing healthcare provision, with NCD prevention made more integral to that from this intervention. The question thus arises: can the strengths in the primary care system be harnessed, as occurred in this intervention, to reduce CVD risk factors and thus CVD mortality? The results reported here provide evidential support for a Sri Lankan designed, community-based, whole population intervention approach for reducing CVD and T2DM risk factors, implemented by primary care workers with strong community links to local ‘change agents’.

While evaluations of individual-based health promotion interventions to lower NCD risk factors have been frequent in developed countries, evidence is emerging from corresponding population-based interventions to prevent NCD in LMIC settings. A systematic review of controlled trials of health promotion interventions to reduce NCD risk factors led by community health workers (CHW) in LMICs, conducted in Egypt, India, Iran, Pakistan, Thailand, China, Nigeria, Ghana, South Africa, Costa Rica, Mexico and American Samoa, showed significant reductions in SBP and DBP in individuals followed longitudinally [44]. Of the seventeen trial studies included in the review, six targeted the general community and 11 targeted high-risk populations. Four of those targeting the general population had BP, fasting glucose, BMI or total cholesterol outcomes [45–48]. Interventions were conducted by community health workers and/or primary care physicians, one of which incorporated a 2 × 2 factorial study design of CHWs and/or physicians [48]. For BP, longitudinal changes in intervention versus control groups favoured the intervention groups for SBP, ranging from − 9 mmHg to − 0.89 mmHg, but not all were statistically significant. Results were somewhat different for DBP, ranging from +2.22 mmHg to − 3.95 mmHg, with some statistically significant and the positive DBP difference coming from a single study. Other studies of community-based health promotion interventions not included in the review also showed similar results in favour of the intervention, for example that of Jafar et al. [2009] [49].

While our results for SBP and DBP are of a somewhat similar magnitude to these studies, they are not directly comparable since they are cross-sectional differences (in different individuals) before-and-after the intervention rather than differences in longitudinal changes (in the same individuals) between those contemporaneously exposed and not exposed to the interventions.

The approach, where differences in population CVD risk factors from STEPS or similar surveys of different individuals from the same population conducted at different time points, is used in to infer secular changes in CVD risk factors in the population overall, is the usual method of assessment of population interventions for primary prevention of CVD, and is implicit in the WHO intermittent STEPS methodology.

Comparable programs and interventions implemented in the WHO Western Pacific region targeting community prevention and control of NCD have had varying degrees of success [50–52]. Schultz et al. [2011], in their review of a community-based intervention to address obesity in the Pacific, argued that local leadership and ownership, and having skills and infrastructure available locally, were critical to the success of community-based interventions, and their absence a reason for failure in past programs [52]. The present study did not impose a pre-designed approach from elsewhere, which adds weight to the findings of Schultz et al.

Several NCD prevention programs have targeted CVD and T2DM risk factors in Sri Lanka over the last decade. The HLCs, comprising > 900 centres by 2018, [30] mostly are attached to hospitals, with some attached to community health facilities. No HLCs were located within the study area of the present study. The effectiveness of HLCs in reduction of risk factors in the surrounding population is unclear and needs evaluation.

The maintenance and continued reduction in CVD risk factors found in the present study will depend on the extent that community-based interventions become embedded in the PHC system and local behavioural culture. Attribution of the observed changes evidenced in the present study would be enhanced by a Stepped Wedge study design [53].

A strength of this study is that it consists of a defined population, and that the baseline and follow-up surveys (sampling fractions 11–12%) were from randomly selected villages/settlements providing different subjects minimising possible Hawthorne effects from previous testing [54]. Furthermore, the unexposed group was truly unexposed to the intervention because the intervention had not occurred when surveyed. Thus contamination from the intervention in control groups was precluded which is always possible in contemporaneous exposed and unexposed comparisons. A disadvantage of the study is the lack of non-intervention control areas for contemporaneous comparison. However, this study was designed to pre-test a community primary health care intervention using existing staff, which could be then assessed in a larger cluster randomised investigation including risk factor testing and control arms using a Stepped Wedge design.

To determine that the findings before and after the interventions were not due to significantly differing samples, the analyses were repeated for only those villages randomly selected for both surveys. Results from these 31 villages (*n*=1,520) showed pre- and post-intervention hypertension prevalences to be within 1–2 percentage points of corresponding estimates from the total sample (cf. Table 2), with similar statistically significant differences pre- and post-intervention.

A strength of the community health promotion intervention was its design and implementation by the co-authors and regional health services, with consultation and input from local communities, using existing and available primary care staff, with a focus on women. A significant failure of the intervention was the lack of change in the prevalence of tobacco smoking in men. As tobacco smoking in Sri Lankan women remains extremely low, a sex-specific tobacco control strategy aimed specifically at men needs to be included in future initiatives which could involve local environmental health officers and health inspectors (including a significant proportion of men), with focus on workplaces as sites for health promotion activities.

The slightly higher mean and median BMI, and odds ratios for obesity prevalence, in the second survey compared to the first, were either not statistically significant, or became non-significant after adjustment for education and income, suggesting minor differences between samples; these findings cannot be further interpreted in view of the small magnitude of differences and lack of statistical significance.

Although an unexposed control group to compare contemporaneously with the intervention population before and after the intervention would have been useful, plausible causes of changes of such magnitude occurring over a three-year intervening period are not evident, especially given that no HLCs existed in the study areas at the time of the first or second survey.

### Conclusions

Following three years of community health promotion initiatives and activities implemented through primary health care in four PHM areas in the Kalutara district of Sri Lanka, population means and prevalences of CVD risk factors, including hypertension and blood pressures, diabetes and fasting plasma glucose, and hypercholesterolaemia and total blood cholesterol, were significantly lower in a separate sample of adults in the study area. Reducing male tobacco smoking and BMI were unchanged and remain healthcare challenges in Sri Lanka.

The main factors behind the success of this intervention that could be replicated include: local health service development and ownership of the intervention, with appropriate technical collaborations; a strong pre-existing and authoritative primary health care network to initiate and integrate NCD prevention into routine healthcare provision; use of influential ‘change agents’ to maximise the chances of the messages of the intervention influencing health behaviours that target as many differing segments of the population as is feasible.

## Appendix

## Data Availability

The datasets generated during and/or analysed during the current study are not publicly available, due to the potential of compromising participant privacy, but are available from the corresponding author on reasonable request.

## Abbreviations

M: Male (or men)
F: Female (or women)
CVD: Cardiovascular disease
T2DM: Type 2 diabetes
SBP: Systolic blood pressure
DBP: Diastolic blood pressure
FPG: Fasting plasma glucose
HLC: Healthy lifestyle centre
CHW: Community health worker
PHCW: Primary healthcare worker
LMIC: Low and middle income country
WHO: World Health Organization
